# Associations of *ACE I/D* and *AGTR1* rs5182 polymorphisms with diabetes and their effects on lipids in an elderly Chinese population

**DOI:** 10.1186/s12944-024-02222-w

**Published:** 2024-07-30

**Authors:** Jun Yi Liu, Yan Zhi Yi, Qi Wei Guo, Ke Xin Jia, Xue Cheng Li, Jia Jing Cai, Yi Lin Shen, Guo Ming Su, Xu Chen, Xing Yu Zhang, Ding Zhi Fang, Hao Hong, Jia Lin

**Affiliations:** 1 Department of Biochemistry and Molecular Biology, West China School of Basic Medical Sciences & Forensic Medicine, Sichuan University, Chengdu, 610041 P. R. China; 2 Department of Spinal Surgery, Chongqing Orthopedic Hospital of Traditional Chinese Medicine, Chongqing, P. R. China; 3 Department of Orthopaedic Surgery, Second Affiliated Hospital of Chongqing Medical University, Chongqing, P. R. China

**Keywords:** Diabetes mellitus, *ACE I/D* polymorphism, *AGTR1* rs5182 polymorphism, Dyslipidaemia, Combination analysis

## Abstract

**Background:**

Diabetes mellitus is generally accompanied by dyslipidaemia, but inconsistent relationships between lipid profiles and diabetes are noted. Moreover, genetic variations in insertion/deletion (I/D) polymorphisms at angiotensin-converting enzyme gene (*ACE*) and T/C polymorphisms in the angiotensin type 1 receptor gene (*AGTR1*) are related to diabetes and lipid levels, but the associations are controversial. Thus, the current research aimed to explore the effects of *ACE I/D*, *AGTR1* rs5182 and diabetes mellitus on serum lipid profiles in 385 Chinese participants with an average age of 75.01 years.

**Methods:**

The *ACE I/D* variant was identified using the polymerase chain reaction (PCR) method, whereas the *AGTR1* rs5182 polymorphism was identified using the PCR-based restriction fragment length polymorphism (PCR-RFLP) method and verified with DNA sequencing. Total cholesterol (TC), triglyceride (TG), apolipoprotein A (ApoA), apolipoprotein B (ApoB), high-density lipoprotein cholesterol (HDL-C) and low-density lipoprotein cholesterol (LDL-C) levels were measured using routine methods, and the lipid ratios were calculated.

**Results:**

*ACE I/D*, but not *AGTR1* rs5182, was a predictor of TG/HDL-C for the whole study population. Both *ACE I/D* and *AGTR1* rs5182 were predictors of HDL-C and LDL-C levels in females but not in males. Moreover, in females, diabetes mellitus and *ACE I/D* were identified as predictors of TG and TG/HDL-C, whereas *AGTR1* rs5182 and diabetes mellitus were predictors of TG/HDL-C. Moreover, diabetes mellitus and the combination of *ACE I/D* and *AGTR1* rs5182 variations were predictors of TG and TG/HDL-C exclusively in females.

**Conclusions:**

The results demonstrated the potential for gender-dependent interactions of *ACE I/D*, *AGTR1* rs5182, and diabetes on lipid profiles. These findings may serve as an additional explanation for the inconsistent changes of blood lipids in individuals with diabetes mellitus, thereby offering a novel perspective for the clinical management of blood lipid levels in diabetic patients.

**Supplementary Information:**

The online version contains supplementary material available at 10.1186/s12944-024-02222-w.

## Background

Diabetes mellitus has become an expanding public health concern globally, affecting more than 37% of individuals aged 65 years and older [1]. Moreover, diabetes mellitus is generally accompanied by dyslipidaemia [2], the typical features of which include elevated triglyceride (TG) and low-density lipoprotein cholesterol (LDL-C) levels as well as decreased high-density lipoprotein cholesterol (HDL-C) levels [3]. Elevated TG levels and reduced HDL-C levels are often accompanied by diabetes mellitus [4]. In addition, as a sensitive parameter reflecting blood lipid profiles, the TG-to-HDL-C ratio (TG/HDL-C) is significantly positively associated with diabetes mellitus [5]. However, inconsistent relationships between serum lipids and diabetes mellitus have also been reported. For example, TG is associated with a reduced risk of diabetes mellitus in patients who exhibit genetic susceptibility to elevated TG levels [6]. Similarly, the protective effect of HDL for diabetes was found only in Iranian women but not in Iranian men [7]. Thus, the critical effects of genetic background should be considered in the context of the complex relationship between lipid fractions and diabetes mellitus.

The renin‒angiotensin system (RAS), a complex hormonal regulatory system, is associated with not only blood pressure but also dyslipidaemia [8]. Interfering with the RAS using drugs has become a type of diabetes treatment [9]. In the RAS, angiotensin-converting enzyme (ACE) can convert angiotensin I to angiotensin II through the removal of carboxy-terminal dipeptides [8]. The significant effects of ACE on glycaemic disturbances suggest a correlation between ACE and diabetes mellitus [10], which was further confirmed by the utilization of ACE inhibitors to prevent diabetes mellitus [11]. The ACE gene (*ACE*), which is located on the long arm of chromosome 17 (17q23), is 21 kb long and consists of 26 exons and 25 introns [12]. The *ACE I/D* polymorphism is distinguished by a distinctive 287-base pair repetitive element located within intron 16, which gives rise to the variation of either an insertion or a deletion (I/D) within the genetic sequence [13]. This variation leads to 3 possible genotypes, including II, ID and DD [13], as well as a change in ACE concentration [14]. Previous studies have shown that *ACE I/D* is associated with diabetes mellitus [15], and II homozygotes had higher HDL-C levels than did subjects with the D allele [13, 16]. On the other hand, other studies noted that the association between the *ACE I/D* polymorphism and dyslipidaemia was insignificant in Chinese diabetic patients [17, 18]. Obviously, other factors should be considered to explain the conflicting findings regarding the influence of *ACE I/D* on lipid profiles in diabetic patients.

As the major biologically active hormone generated by the RAS system, angiotensin II regulates blood pressure via angiotensin type 1 receptor (AGTR1) [8, 19]. The human AGTR1 gene (*AGTR1*), which contains 5 exons and 4 introns, is located on the long arm of chromosome 3 (3q21-25) [20]. The rs5182 polymorphism (C573T) of *AGTR1* alters *AGTR1* expression [21] and is related to the presence of diabetes combined with hypertension in the Han population of Inner Mongolia [20]. The *AGTR1* rs5182 variant is related to nonalcoholic fatty liver disease, which is often characterized by dyslipidaemia [22, 23]. However, a study conducted in another Chinese population failed to observe a significant association between *AGTR1* rs5182 and dyslipidaemia [21]. Thus, the effects of *ACE I/D* and *AGTR1* rs5182 on lipid profiles, as well as their interaction with diabetes mellitus, should be studied to obtain a better understanding about the influence of diabetes and genetic polymorphisms on lipids.

This study seeks to explore possible explanations for the controversial findings concerning the relationship between diabetes and dyslipidaemia reported in previous studies and to evaluate the cumulative effect of *ACE I/D* and *AGTR1* rs5182 on lipid levels, which has not yet been reported. It is hypothesized that interactions potentially occur among *ACE I/D* variation, the *AGTR1* rs5182 polymorphism and diabetes mellitus to impact serum lipid profiles in the current study. Therefore, *ACE I/D* variation, the *AGTR1* rs5182 polymorphism and serum lipid levels were measured in 385 Chinese subjects with an average age of 75.01 years. The interactions between genetic backgrounds and diabetes mellitus, as well as their contributions to lipid profiles, were analysed. The investigation of the combined effects of *ACE I/D* and *AGTR1* rs5182 on lipid profiles, as well as their interactions with diabetes mellitus in the current study, may contribute to preventing and managing dyslipidaemia in individuals with diabetes mellitus.

## Methods

### Study population

One thousand one hundred nineteen volunteers were enrolled, and the inclusion criteria for the participants were as follows: (1) understood the procedures involved and provided written consent; (2) had a history of diabetes; (3) provided current medication use status for antidiabetic and/or lipid-lowering drugs; (4) provided complete serum lipid and glucose measurements; (5) provided blood samples; and (6) were aged ≥ 50 years. In total, 385 of the participants (average age = 75.01 ± 24.90 years) who met the above criteria were involved in the present study. The study was approved by the Human Research Ethics Committee of Chongqing Orthopedic Hospital of Traditional Chinese Medicine.

### Biochemical measurements

Venous blood samples were collected from the participants in the morning after a 12-hour fast. Serum was isolated via centrifugation (3000 rpm, 20 min) at 4 °C and stored at -80 °C for further analyses. Glucose levels were measured using the glucose oxidase‒peroxidase (GOD‒POD) method [24]. Serum TG levels were determined using the glycerol phosphate oxidase-p-aminophenazone (GPO-PAP) method [25]. TC, HDL-C and LDL-C levels were measured using the cholesterol oxidase-peroxidase and 4-aminoantipyrine phenol (CHOD-PAP) method [25]. Apolipoprotein A (ApoA) and apolipoprotein B (ApoB) levels were determined via immunoturbidimetry [26]. Ratios of TG/HDL-C, TC/HDL-C and LDL-C/HDL-C were calculated.

### DNA extraction and genotyping

Genomic DNA was extracted using a DNA extraction kit per the manufacturer’s instructions (Kuang Yuan, Suzhou, China). The *ACE I/D* genotype was detected via the polymerase chain reaction (PCR) method, and the *AGTR1* rs5182 variant was identified via the polymerase chain reaction-restriction fragment length polymorphism (PCR-RFLP) method followed by confirmation using DNA sequencing. Briefly, for *ACE I/D* genotype detection, the target DNA fragments were amplified with the primers 5’-CTGGAGACCACTCCCATCCTTTCT-3’ (forward) and 5’-GATGTGGCCATCACATTCGTCAGA T-3’ (reverse) [13]. The samples were denatured at 94 °C for 4 min, followed by 32 cycles, which consisted of denaturation at 94 °C for 1 min, annealing at 56 °C for 1 min and extension at 72 °C for 90 s, with a final extension at 72 °C for 5 min. A 190-bp PCR fragment was produced in the absence of the insertion (D), and a 490-bp fragment was produced in the presence of the insertion (I). In the absence of the insertion (D), a PCR product of 190 bp was generated, whereas the presence of the insertion (I) resulted in the amplification of a 490-bp fragment. Moreover, for *AGTR1* rs5182 genotype identification, two oligonucleotide primers, 5’-GGCTTTGCTTTGTCTTGTTG-3’ (forward) and 5’-AATGCTTGTAGCCAAAGTCACCT-3’ (reverse), were used for amplification [27]. The PCR procedure consisted of 3 min at 94 °C for denaturation; 40 cycles of 30 s at 94 °C, 30 s at 60 °C, and 90 s at 72 °C; and a final elongation step of 5 min at 72 °C [27]. The PCR-amplified products were digested overnight with the restriction endonuclease *MnI*I, which cuts at position 580, when the C allele is present instead of the T allele at 573, and at positions 905, 1032, 1062, and 1147. The PCR-amplified products of *ACE I/D* and the restriction endonuclease *MnI*I-digested products of *AGTR1* rs5182 were identified using 1.5% agarose gel electrophoresis and verified with DNA sequencing.

### Dummy variable coding

Owing to the limited number, the minor allele homozygotes were combined with their heterozygotes and defined as D allele carriers of *ACE I/D* and C allele carriers of *AGTR1* rs5182 for further analysis. To further clarify the cumulative effects of *ACE I/D* and *AGTR1* rs5182 on lipid levels, the combined genotypes of *ACE I/D* and *AGTR1* rs5182 were used as dummy variables:

$$Dummy{\text{ }}variable\,1 = ACEII{\text{ }} + AGTR1\,rs5182{\text{ }}TT$$

$$Dummy{\text{ }}variable{\text{ }}2 = ACEII + AGTR1\,rs5182{\text{ }}C{\text{ }}allele$$

$$Dummy{\text{ }}variable{\text{ }}3{\text{ }} = ACED{\text{ }}allele + AGTR1\,rs5182{\text{ }}TT$$

$$Dummy{\text{ }}variable{\text{ }}4{\text{ }} = ACED{\text{ }}allele\,+\,AGTR1\,rs5182{\text{ }}C{\text{ }}allele$$

### Statistical analyses

The data are expressed as the means ± standard deviations (SDs) unless otherwise specified. The sample size calculation was conducted using the G*Power software program (version 3.1.9.7, Germany) [28], and the current sample size was sufficient for a minimum power of 80%. The deviation from Hardy‒Weinberg equilibrium [29], which can estimate the number of homozygous and heterozygous variant vectors in an unevolved population [30], was analysed with the χ^2^ goodness-of-fit test. The chi-square test was used to determine the distribution of genotypes and alleles, the prevalence of diabetes and the percentage of drug use between subjects of different genders. The normal distribution of each variable was initially analysed using the Kolmogorov-Smirnov test. Because of the abnormal distribution, logarithmic transformations were applied to TG and the TG/HDL-C ratio to reduce skewness before performing the statistical analyses. Independent sample t tests were conducted to compare the differences in blood lipid levels between the males and the females. Potential factors associated with blood lipid and blood glucose levels were analysed using the stepwise multiple linear regression analysis. Statistical significance was defined as *P <* 0.05.

## Results

### Characteristics of the study population

Table 1 shows the characteristics of the metabolic variables, prevalence of diabetes, and drug usage in the current study population. As displayed in Table 1, TG, TC, HDL-C, ApoA, LDL-C, and ApoB levels as well as the prevalence of diabetes were significantly greater in females than in males (*P* = 0.017, *p* = 0.016, *P* = 0.049, *P* = 0.002, *P* = 0.021, *P* = 0.022, and *P* = 0.045, respectively). However, no statistically significant differences in age or glucose were noted between the genders. When considering the use of lipid-lowering drugs and treatment with antidiabetic drugs, no significant difference was found between male and female subjects.

**Table 1:** Characteristics of the study population

Variables	All	Males	Females
N	385	229	156
Age^a^, year	75.01 ± 24.90	77.00 ± 31.00	72.00 ± 10.00
TG^a^, mmol/L	1.68 ± 1.20	1.61 ± 1.22	1.78 ± 1.16^*^
TC^a^, mmol/L	3.48 ± 1.55	3.32 ± 1.60	3.70 ± 1.44^*^
HDL-C^a^, mmol/L	0.91 ± 0.40	0.88 ± 0.38	0.96 ± 0.41^*^
ApoA^a^, mmol/L	1.08 ± 0.36	1.03 ± 0.36	1.14 ± 0.36^**^
LDL-C^a^, mmol/L	1.86 ± 1.00	1.76 ± 1.10	2.00 ± 0.83^*^
ApoB^a^, mmol/L	0.71 ± 0.30	0.68 ± 0.32	0.75 ± 0.28^*^
Glucose^a^, mmol/L	7.18 ± 3.06	7.32 ± 3.13	6.97 ± 2.95
Diabetes mellitus^b^, n (%)			
With	114(30.0)	59(25.8)	55(35.3) ^&^
Without	271(70.0)	170(74.2)	101(64.7)
Lipid-lowering drugs^b^, n (%)			
Yes	81(21.0)	49(21.4)	32(20.5)
No	304(79.0)	180(78.6)	124(79.5)
Antidiabetic drugs^b^, n (%)			
Yes	138(36.0)	82(35.8)	56(35.9)
No	247(64.0)	147(64.2)	100(64.1)

^a^Data are expressed as mean ± SD or median; ^b^Data are presented as n (%)

TG, triglycerides; TC, total cholesterol, HDL-C, high-density lipoprotein cholesterol; ApoA: apolipoprotein A; LDL-C, low-density lipoprotein cholesterol; ApoB: apolipoprotein B

^*^P < 0.05, and ^**^P ≤ 0.01, comparisons between the male subjects and the female subjects (independent-samples t test); ^&^*P* < 0.05, comparisons between the male subjects and the female subjects (Chi-square tests)

### Genotypes and alleles of *ACE I/D* and *AGTR1* rs5182 in the participants

**Fig. 1:**
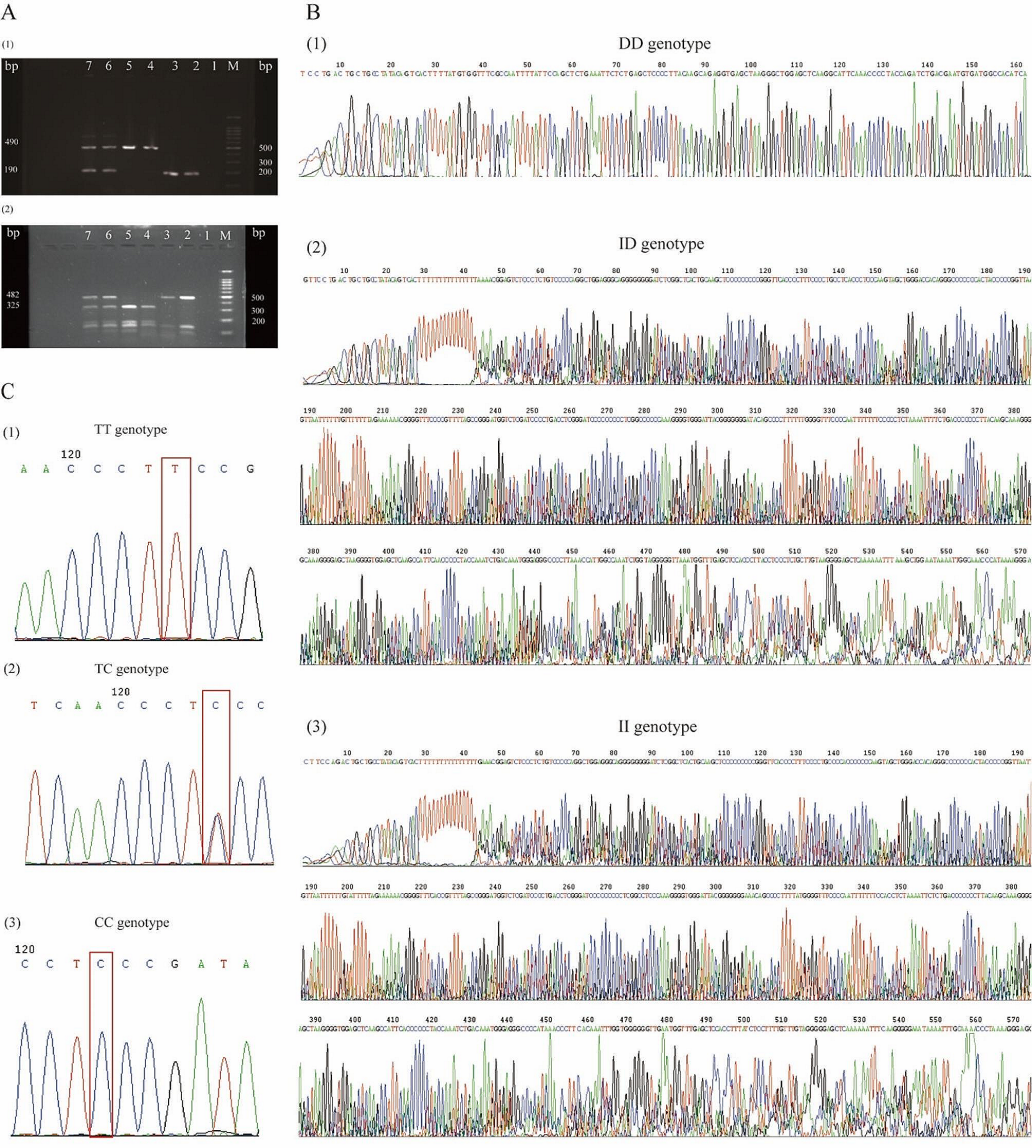
Gel images of *ACE I/D* and *AGTR1* rs5182 genotyping and sequencing results. **(A)** Gel images of *ACE I/D* and rs5182 genotyping: (1) gel images of *ACE I/D* genotyping, M: DNA ladder, 1: blank, 2/3: DD, 4/5: II, 6/7: ID; (2) gel images of *AGTR1* rs5182 genotyping, M: DNA ladder, 1: blank, 2/3: TT, 4/5: CC, 6/7: TC. **(B)** Sequencing results of the *ACE I/D* (1) DD genotype, (2) ID genotype, and (3) II genotype. **(C)** Sequencing results for *AGTR1* rs5182: (1) TT genotype, (2) TC genotype, (3) CC genotype

**Fig. 2:**
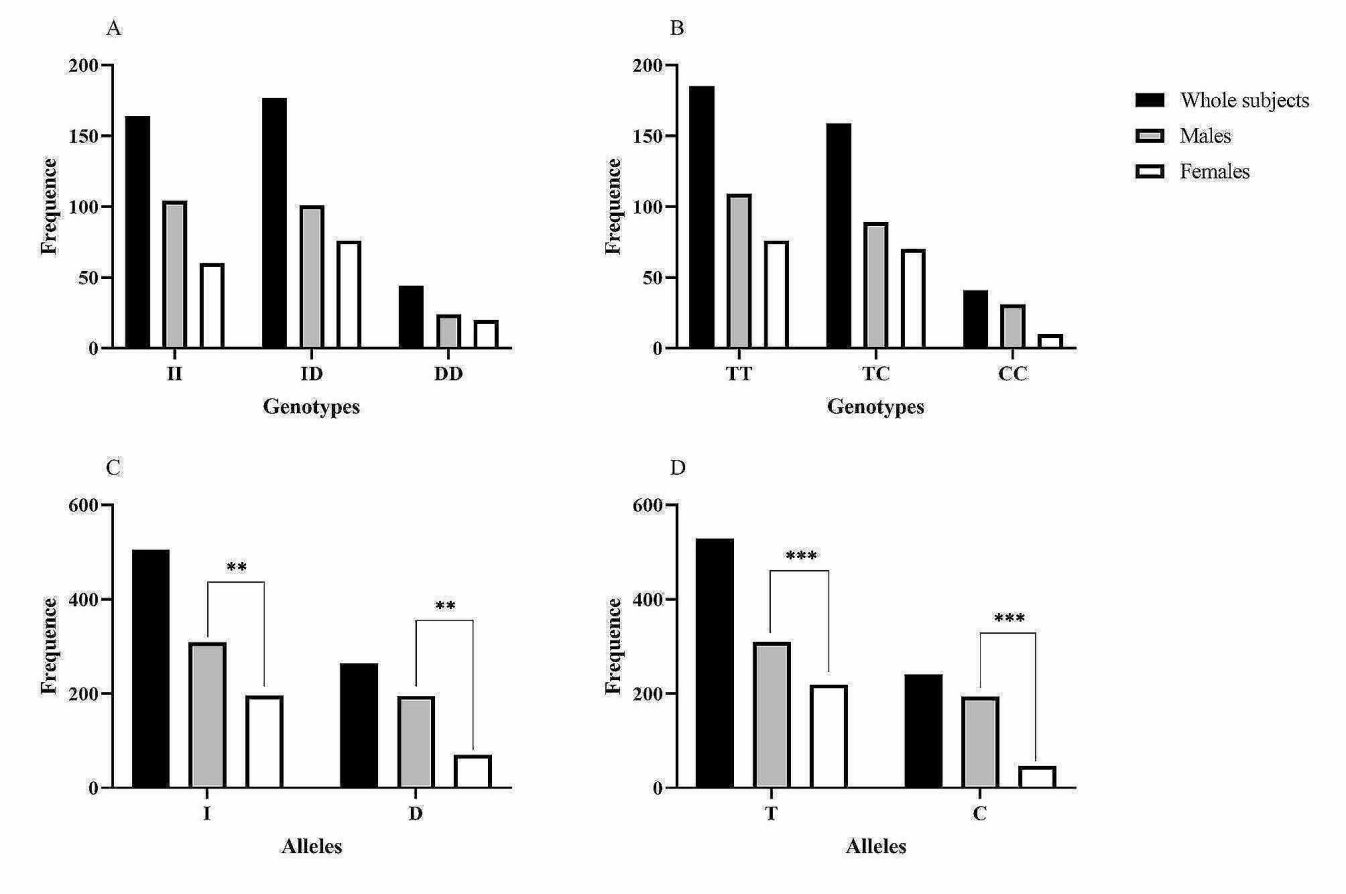
Frequencies of genotypes and alleles of *ACE I/D* and *AGTR1* rs5182 in the study population. **(A)** genotype frequence of *ACE I/D*. **(B)** genotype frequence of *AGTR1* rs5182. **(C)** Allele frequence of *ACE I/D*; **(D)** Allele frequence of *AGTR1* rs5182. ^**^P ≤ 0.01, ^***^P < 0.001

The identification of *ACE I/D* and *AGTR1* rs5182 using gel electrophoresis, followed by confirmation via DNA sequencing, is shown in Fig. 1. The frequencies of genotypes and alleles are presented in Fig. 2. The genotype frequencies of either *ACE I/D* or *AGTR1* rs5182 were in Hardy‒Weinberg equilibrium in the current study (*P* = 0.820 and *P* = 0.480, respectively). No statistically significant differences in the genotype frequencies of the *ACE I/D* genotype and *AGTR1* rs5182 genotype were noted between male and female subjects. However, the D allele of *ACE I/D* and C allele of *AGTR1* rs5182 exhibited significantly greater frequencies in males compared with females. Owing to the limited numbers of samples, the minor allele homozygotes were combined with their heterozygotes and defined as D allele carriers of *ACE I/D* and C allele carriers of *AGTR1* rs5182, respectively, for further analysis.

### Predictors of lipid levels in the subjects

To further explore the predictors of lipid profiles in the current study population, stepwise multiple linear regression analyses were performed. The *ACE I/D* genotype (Model A) or *AGTR1* rs5182 genotype (Model B) was used as an independent variable separately or in combination with age, gender, diabetes mellitus, and the use of antidiabetic drugs or lipid-lowering drugs as other independent variables.

As shown in Table 2, gender was the only predictor of TG, TC, and ApoB levels, explaining 1.2%, 1.5%, and 1.4% of the total variance, respectively. Gender, antidiabetic drug use, and lipid-lowering drug use were predictors of HDL-C levels, accounting for 2.7%, 1.1% and 0.8% of the total variance, respectively. In addition, gender, the use of antidiabetic drugs, lipid-lowering drugs and diabetes mellitus were predictors of ApoA levels, accounting for 2.3%, 3.2%, 2.3%, and 0.9% of the total variance, respectively. Moreover, gender, the use of antidiabetic drugs, and diabetes mellitus were predictors of LDL-C levels, accounting for 0.9%, 1.3% and 1.2% of the total variance, respectively. The use of antidiabetic drugs, diabetes mellitus status and age were predictors of glucose levels, accounting for 12.3%, 2.8% and 2.3% of the total variance, respectively. Notably, *ACE I/D* and the use of antidiabetic drugs were predictors of the TG/HDL-C ratio, accounting for 1.0% and 1.6% of the total variance, respectively. However, *AGTR1* rs5182 was not a predictor of TG/HDL-C.

**Table 2:** Predictor of lipid profiles in the whole subjects

Dependent Variables	Independent Variables	Model A^a^		Model B^b^	
		β	Partial correlation	β	Partial correlation
TG	Gender	0.122^*^	0.122	0.122^*^	0.122
TC	Gender	0.122^*^	0.122	0.122^*^	0.122
HDL-C	Gender	0.102^*^	0.104	0.102^*^	0.104
	Antidiabetic drugs	-0.180^***^	-0.182	-0.180^***^	-0.182
	Lipid-lowering drugs	0.117^*^	0.119	0.117^*^	0.119
ApoA	Gender	0.145^**^	0.150	0.145^**^	0.150
	Antidiabetic drugs	-0.244^***^	-0.230	-0.244^***^	-0.230
	Lipid-lowering drugs	0.159^**^	0.165	0.159^**^	0.165
	Diabetes mellitus	0.119^*^	0.114	0.119^*^	0.114
LDL-C	Gender	0.106^*^	0.107	0.106^*^	0.107
	Antidiabetic drugs	-0.170^**^	-0.159	-0.170^**^	-0.159
	Diabetes mellitus	0.118^*^	0.110	0.118^*^	0.110
ApoB	Gender	0.117^*^	0.117	0.117^*^	0.117
TG/HDL-C	Genotype	-0.111^*^	-0.112	-	-
	Antidiabetic drugs	0.136^**^	0.137	0.135^**^	0.135
TC/HDL-C	-	-	-	-	-
LDL-C/HDL-C	-	-	-	-	-
Glucose	Antidiabetic drugs	0.268^***^	0.263	0.268^***^	0.263
	Diabetes mellitus	0.203^***^	0.202	0.203^***^	0.202
	Age	0.159^**^	0.172	0.159^**^	0.172

^a^Gender, age, antidiabetic drugs, lipid-lowering drugs, diabetes mellitus, and the *ACE I/D* genotypes were included as independent variables. ^b^*AGTR1* rs5182 genotypes, not *ACE I/D* genotypes were included as independent variables, and all the other independent variables were the same as Model A. TG, TC, ApoA, ApoB, HDL-C, LDL-C, TG/HDL-C, TC/HDL-C, LDL-C/HDL-C and glucose were included as dependent variable

β, Standardized regression coefficient; -, variable not entered in the stepwise multiple linear regression model

TG, triglyceride; TC, total cholesterol; HDL-C, high-density lipoprotein cholesterol; ApoA, apolipoprotein A; LDL-C; low-density lipoprotein cholesterol; ApoB, apolipoprotein B

^*^P < 0.05, ^**^P ≤ 0.01, and ^***^P < 0.001

When gender was taken into consideration in the *ACE I/D* analysis, as displayed in Table 3, the use of antidiabetic drugs, the use of lipid-lowering drugs and diabetes mellitus were predictors of HDL-C (accounting for 2.4%, 2% and 2.8% of the total variance, respectively) and ApoA (accounting for 4.3%, 3.4%, and 3.6% of the total variance, respectively) levels in males. The use of antidiabetic drugs and diabetes mellitus were predictors of LDL-C levels, contributing 2.7% and 1.6% of the total variance, respectively, whereas the use of antidiabetic drug was a predictor of ApoB levels in males, contributing 2.4% of the total variance. Furthermore, the use of antidiabetic drugs, diabetes mellitus, and age were predictors of glucose levels in males, contributing to 12.3%, 2.1% and 2.3% of the total variance, respectively. Although *ACE I/D* was not a predictor of any lipid profile in males, notably, in female subjects, *ACE I/D* and diabetes mellitus were predictors of TG (accounting for 3.7% and 3.5% of the total variance, respectively) and TG/HDL-C (accounting for 4.3% and 3.9% of the total variance, respectively). Moreover, age was the only predictor of ApoA levels, and *ACE I/D* was the only predictor of LDL-C levels in females, accounting for 3.6% and 4.4% of the total variance, respectively. In terms of HDL-C levels in females, *ACE I/D*, age and the use of antidiabetic drugs were predictors, accounting for 2.9%, 2.7% and 2.3% of the total variance, respectively. The predictors of glucose in females were the same as those in identified males, including the use of antidiabetic drugs, diabetes mellitus, and age, contributing to 2.8%, 14% and 1.9% of the total variance, respectively.

**Table 3:** Predictor of lipid profiles in subjects with different *ACE I/D* genotype in males and females

Dependent Variables	Males (*n* = 229)				Females (*n* = 156)		
	Independent Variables	β	Partial correlation		Independent Variables	β	Partial correlation
TG	-	-	-		Diabetes mellitus	0.219^**^	0.222
	-	-	-		*ACE I/D* ^a^	-0.209^**^	-0.213
TC	-	-	-		-	-	-
HDL-C	Antidiabetic drugs	-0.237^***^	-0.230		*ACE I/D* ^a^	0.179^*^	0.185
	Lipid-lowering drugs	0.157^*^	0.161		Age	-0.174^*^	-0.180
	Diabetes mellitus	0.187^**^	0.184		Antidiabetic drugs	-0.169^*^	-0.175
ApoA	Antidiabetic drugs	-0.297^***^	-0.290		Age	-0.205^*^	-0.205
	Lipid-lowering drugs	0.198^**^	0.206		-	-	-
	Diabetes mellitus	0.205^**^	0.205		-	-	-
LDL-C	Antidiabetic drugs	-0.219^**^	-0.210		*ACE I/D* ^a^	0.210^**^	0.210
	Diabetes mellitus	0.148^*^	0.144		-	-	-
ApoB	Antidiabetic drugs	-0.168^*^	-0.168		-	-	-
TG/HDL-C	-	-	-		Diabetes mellitus	0.230^**^	0.234
	-	-	-		*ACE I/D* ^a^	-0.222^**^	-0.226
TC/HDL-C	-	-	-		*-*	-	-
LDL-C/HDL-C	-	-	-		*-*	-	-
Glucose	Antidiabetic drugs	0.294^***^	0.295		Antidiabetic drugs	0.211^*^	0.199
	Diabetes mellitus	0.180^**^	0.186		Diabetes mellitus	0.265^**^	0.247
	Age	0.165^**^	0.178		Age	0.155^*^	0.171

Age, antidiabetic drugs, lipid-lowering drugs, diabetes mellitus, and *ACE I/D* were included as independent variables. TG, TC, ApoA, ApoB, HDL-C, LDL-C, TG/HDL-C, TC/HDL-C, LDL-C/HDL-C and glucose were included as dependent variable

β, Standardized regression coefficient; -, not included as an independent variable

TG, triglyceride; TC, total cholesterol; HDL-C, high-density lipoprotein cholesterol; ApoA, apolipoprotein A; LDL-C; low-density lipoprotein cholesterol; ApoB, apolipoprotein B

^a^1 = ID/DD genotype, 2 = II genotype

^*^P < 0.05, ^**^P ≤ 0.01, and ^***^P < 0.001

The results of the analysis of the effects of *AGTR1* rs5182 in males and females are shown in Table 4. The predictors of HDL-C, ApoA, LDL-C, ApoB, and glucose levels, as well as the contribution of each factor to the total variance in males, were the same as those identified in the *ACE I/D* analysis (Table 3). Similarly, the predictors of glucose levels and their contributions to the total variance in females were the same as those identified in the *ACE I/D* analysis (Table 3). Nevertheless, in female subjects, diabetes mellitus was the only predictor of TG levels, whereas age was the only predictor of ApoA levels, accounting for 3.5% and 3.6%, respectively, of the total variance. Interestingly, *AGTR1* rs5182, age and the use of antidiabetic drugs were predictors of HDL-C levels in females, accounting for 2.9%, 2.3%, and 2.2%, of the total variance, respectively. The *AGTR1* rs5182 was the only predictor of LDL-C levels in females, accounting for 2.0% of the total variance. Furthermore, *AGTR1* rs5182 and diabetes mellitus were predictors of TG/HDL-C, accounting for 2.4% and 3.9% of the total variance, respectively.

**Table 4:** Predictor of lipid profiles in subjects with different *AGTR1* rs5182 genotype in males and females

Dependent Variables	Males (*n* = 229)				Females (*n* = 156)		
	Independent Variables	β	Partial correlation		Independent Variables	β	Partial correlation
TG	-	-	-		Diabetes mellitus	0.202^*^	0.202
TC	-	-	-		-	-	-
HDL-C	Antidiabetic drugs	-0.237^***^	-0.230		*AGTR1* rs5182^a^	0.166^*^	0.172
	Lipid-lowering drugs	0.157^*^	0.161		Age	-0.184^*^	-0.190
	Diabetes mellitus	0.187^**^	0.184		Antidiabetic drugs	-0.170^*^	-0.175
ApoA	Antidiabetic drugs	-0.297^***^	-0.290		Age	-0.205^*^	-0.205
	Lipid-lowering drugs	0.198^**^	0.206		-	-	-
	Diabetes mellitus	0.205^**^	0.205		-	-	-
LDL-C	Antidiabetic drugs	-0.219^**^	-0.210		*AGTR1* rs5182^a^	0.163^*^	0.163
	Diabetes mellitus	0.148^*^	0.144		-	-	-
ApoB	Antidiabetic drugs	-0.168^*^	-0.168		-	-	-
TG/HDL-C	-	-	-		Diabetes mellitus	0.204^*^	0.207
	-	-	-		*AGTR1* rs5182^a^	-0.174^*^	-0.177
TC/HDL-C	-	-	-		*-*	-	-
LDL-C/HDL-C	-	-	-		*-*	-	-
Glucose	Antidiabetic drugs	0.294^***^	0.295		Antidiabetic drugs	0.211^*^	0.199
	Diabetes mellitus	0.180^**^	0.186		Diabetes mellitus	0.265^**^	0.247
	Age	0.165^**^	0.178		Age	0.155^*^	0.171

Age, antidiabetic drugs, lipid-lowering drugs, diabetes mellitus, and *AGTR1* rs5182 were included as independent variables. TG, TC, ApoA, ApoB, HDL-C, LDL-C, TG/HDL-C, TC/HDL-C, LDL-C/HDL-C and glucose were included as dependent variable

β, Standardized regression coefficient; -, not included as an independent variable

TG, triglyceride; TC, total cholesterol; HDL-C, high-density lipoprotein cholesterol; ApoA, apolipoprotein A; LDL-C; low-density lipoprotein cholesterol; ApoB, apolipoprotein B

^a^1 = TC/CC genotype, 2 = TT genotype

^*^P < 0.05, ^**^P ≤ 0.01, and ^***^P < 0.001

### *ACE I/D* and *AGTR1* rs5182 combination analysis in the subjects

To further investigate the combined influence of the *ACE I/D* polymorphism and the *AGTR1* rs5182 variant on lipid profiles, stepwise multiple linear regression analyses were performed. Dummy variables 1 (*ACE* II + *AGTR1* rs5182 TT), 2 (*ACE* II + *AGTR1* rs5182 C allele), and 4 (*ACE* D allele + *AGTR1* rs5182 C allele) were incorporated as independent variables, whereas dummy variable 3 (*ACE* D allele + *AGTR1* rs5182 TT) served as the reference category. Additionally, age, gender, the prevalence of diabetes mellitus, and the use of antidiabetic drugs or lipid-lowering drugs were included as other independent variables to estimate their individual predictive value for lipid levels, lipid ratios and glucose levels.

In the whole study population, as shown in Table 5, although the predictors of TG, TC, HDL-C, ApoA, ApoB, LDL-C, and glucose levels, as well as the contribution of each factor to the total variance, were the same as those in Table 2, it is interesting to note that dummy variable 1 and the use of antidiabetic drugs, accounting for 0.8% and 1.6% of the total variance, respectively, were predictors of TG/HDL-C.

**Table 5:** Combination analysis for *ACE I/D* and *AGTR1* rs5182 in the whole subjects

Dependent Variables	Independent Variables	β	Partial correlation
TG	Gender	0.122^*^	0.122
TC	Gender	0.122^*^	0.122
HDL-C	Gender	0.102^*^	0.104
	Antidiabetic drugs	-0.180^***^	-0.182
	Lipid-lowering drugs	0.117^*^	0.119
ApoA	Gender	0.145^**^	0.150
	Antidiabetic drugs	-0.244^***^	-0.230
	Lipid-lowering drugs	0.159^**^	0.165
	Diabetes mellitus	0.119^*^	0.114
LDL-C	Gender	0.106^*^	0.107
	Antidiabetic drugs	-0.170^**^	-0.159
	Diabetes mellitus	0.118^*^	0.110
ApoB	Gender	0.117^*^	0.117
TG/HDL-C	Dummy variable 1^a^	-0.106^**^	-0.107
	Antidiabetic drugs	0.135^*^	0.136
TC/HDL-C	-	-	-
LDL-C/HDL-C	-	-	-
Glucose	Antidiabetic drugs	0.268^***^	0.263
	Diabetes mellitus	0.203^***^	0.202
	Age	0.159^**^	0.172

Gender, age, antidiabetic drugs, lipid-lowering drugs, diabetes mellitus, and dummy variable 1, 2, 4 were included as independent variables. TG, TC, ApoA, ApoB, HDL-C, LDL-C, TG/HDL-C, TC/HDL-C, LDL-C/HDL-C, and glucose were included as dependent variable

β, Standardized regression coefficient; -, not included as an independent variable

TG, triglyceride; TC, total cholesterol; HDL-C, high-density lipoprotein cholesterol; ApoA, apolipoprotein A; LDL-C; low-density lipoprotein cholesterol; ApoB, apolipoprotein B

^a^0 = Other genotypes, Dummy variable 1 1 = *ACE* II + *AGTR1* rs5182 TT;

^*^P < 0.05, ^**^P ≤ 0.01, and ^***^P < 0.001

When gender was taken into consideration, as displayed in Table 6, dummy variable 4 was the only predictor of TG levels in males, accounting for 3.8% of the total variance. Dummy variable 4 and diabetes mellitus were identified as predictors of TG levels in females, accounting for 3.8% and 3.7% of the total variance, respectively. None of the dummy variables were predictors of TC, HDL-C, ApoA, LDL-C, ApoB, TG/HDL-C, TC/HDL-C, LDL-C/HDL-C or glucose levels in males. In contrast, dummy variable 1 was the only predictor of TC (accounting for 5.8%), ApoB (accounting for 2.4%) and LDL-C (accounting for 7.7%) levels, whereas dummy variable 1 and age were predictors of ApoA (accounting for 3.3% and 3.6%, respectively) in females. Moreover, the predictors of HDL-C in females included dummy variable 1, age, and the use of antidiabetic drugs, accounting for 8.4%, 3.2%, and 2.4% of the total variance, respectively. Dummy variable 1, dummy variable 4, and diabetes mellitus were predictors of TG/HDL-C in female subjects and accounted for 5%, 1.9%, and 4% of the total variance, respectively.

**Table 6:** Combination analysis for *ACE I/D* and *AGTR1* rs5182 in subjects with different genders

Dependent Variables	Males (*n* = 229)				Females (*n* = 156)		
	Independent Variables	β	Partial correlation		Independent Variables	β	Partial correlation
TG	Dummy variable 4^a^	0.210^**^	0.210		Diabetes mellitus	0.208^**^	0.213
	-	-	-		Dummy variable 4^a^	0.216^**^	0.210
TC	-	-	-		Dummy variable 1^b^	0.253^**^	0.253
HDL-C	Antidiabetic drugs	-0.237^***^	-0.230		Dummy variable 1	0.304^***^	0.314
	Lipid-lowering drugs	0.157^*^	0.161		Age	-0.188^*^	-0.201
	Diabetes mellitus	0.187^**^	0.184		Antidiabetic drugs	-0.170^*^	-0.182
ApoA	Antidiabetic drugs	-0.297^***^	-0.290		Age	-0.210^**^	-0.214
	Lipid-lowering drugs	0.198^**^	0.206		Dummy variable 1^b^	0.198^*^	0.202
	Diabetes mellitus	0.205^**^	0.205		-	-	-
LDL-C	Antidiabetic drugs	-0.219^**^	-0.210		Dummy variable 1^b^	0.288^***^	0.288
	Diabetes mellitus	0.148^*^	0.144		*-*	-	-
ApoB	Antidiabetic drugs	-0.168^*^	-0.168		Dummy variable 1^b^	0.174^*^	0.174
TG/HDL-C	-	-	-		Dummy variable 1^b^	-0.186^*^	-0.185
	-	-	-		Dummy variable 4^a^	0.165^*^	0.165
	-	-	-		Diabetes mellitus	0.218^**^	0.227
TC/HDL-C	-	-	-		*-*	-	-
LDL-C/HDL-C	-	-	-		*-*	-	-
Glucose	Antidiabetic drugs	0.294^***^	0.295		Antidiabetic drugs	0.211^*^	0.199
	Diabetes mellitus	0.180^**^	0.186		Diabetes mellitus	0.265^**^	0.247
	Age	0.165^**^	0.178		Age	0.155^*^	0.171

Age, antidiabetic drugs, lipid-lowering drugs, diabetes mellitus, and dummy variable 1, 2, 4 were included as independent variables. TG, TC, ApoA, ApoB, HDL-C, LDL-C, TG/HDL-C, TC/HDL-C, LDL-C/HDL-C and glucose were included as dependent variable

β, Standardized regression coefficient; -, not included as an independent variable

TG, triglyceride; TC, total cholesterol; HDL-C, high-density lipoprotein cholesterol; ApoA, apolipoprotein A; LDL-C; low-density lipoprotein cholesterol; ApoB, apolipoprotein B

^a^0 = Other genotypes, Dummy variable 4 1 = *ACE* D allele + *AGTR1* rs5182 C allele; ^b^0 = Other genotypes, Dummy variable 1 1 = *ACE* II + *AGTR1* rs5182 TT

^*^P < 0.05, ^**^P ≤ 0.01, and ^***^P < 0.001

## Discussion

Diabetes mellitus is often accompanied by dyslipidaemia [5]. However, inconsistencies in the relationship between diabetes mellitus and dyslipidaemia have been reported [6, 31], and the mechanism of the observed discrepancy remains unclear. Moreover, the RAS has been reported as a critical system in controlling blood pressure. For example, ACE activity has been reported to be involved in changes in the retinoic acid receptor (RAR)/retinoid X receptor (RXR)-peroxisome proliferator-activated receptor (PPAR) signalling pathway and the suppression of cellular retinol-binding protein 1 (CRBP1), ultimately affecting adipocyte homeostasis and blood lipids [32, 33]. Moreover, AGTR1 activation is related to lipid accumulation in both the livers of C57BL/6 mice and in HepG2 cells [34, 35]. Thus, ACE and AGTR1 could be involved in diabetes and dyslipidaemia. The *ACE I/D* polymorphism and *AGTR1* rs5182 polymorphism are associated with lipid levels, but the findings are also contradictory [21, 22]. Therefore, investigating the effects of the interactions of the *ACE I/D* and *AGTR1* rs5182 polymorphisms with diabetes and the subsequent effects on lipid profiles has the potential to elucidate the possible mechanism underlying the inconsistency among genetic variations, diabetes and lipid levels.

In the present study, diabetes mellitus was a predictor of ApoA and LDL-C levels in the whole study population but not a predictor of other lipid profiles or ratios. Moreover, although diabetes mellitus was identified as a predictor of HDL-C, ApoA, LDL-C and glucose, neither *ACE I/D* nor *AGTR1* rs5182 contributed to changes in lipid and lipid ratios in males (Tables 2, 3 and 4). In contrast, both *ACE I/D* and *AGTR1* rs5182 were identified as predictors of HDL-C and LDL-C exclusively in females (Tables 3 and 4). Furthermore, in the present study, *ACE I/D*, not *AGTR1* rs5182, was identified as a predictor of the TG/HDL-C ratio (Table 2), which might be attributable to the increased expression levels and increased activity of ACE in DD homozygotes [36, 37]. In female subjects, diabetes mellitus and *ACE I/D* were predictors of TG and TG/HDL-C levels (Table 3), whereas diabetes mellitus and *AGTR1* rs5182 were predictors of TG/HDL-C ratios (Table 4). Thus, the interactions among *ACE I/D* variation or the *AGTR1* rs5182 polymorphism with gender and diabetes mellitus are likely involved in the heterogeneous relationships between TG metabolism and diabetes.

There are limited data from previous studies concerning the interplay of *ACE I/D* and *AGTR1* rs5182, as well as their associations with diabetes mellitus in terms of lipid profiles in Chinese subjects. These results revealed that the combination of *ACE I/D* and *AGTR1* rs5182 contributed to the TG/HDL-C ratio in the whole study population; TG levels in males; and TC, ApoB, LDL-C, ApoA and HDL-C levels in females (Tables 5 and 6). The association of *ACE I/D* with lipid levels has been inconsistently reported [38, 39]. In addition, a significant correlation was observed between the *AGTR1* rs5186 variant and TG levels, but the mechanism has not yet been fully elucidated [40, 41]. Therefore, the combined effect of *ACE I/D* and *AGTR1* rs5182 on lipid levels in the current study provides valuable insights into the intricate relationships between the RAS and dyslipidaemia.

Previous studies have shown an association between gender and blood lipids. For example, adult females had lower LDL-C levels and higher HDL-C levels compared with adult males [42]. Moreover, the prevalence of TC, TG, and LDL-C at borderline high or greater levels increased with age in females, but it remained stable or even decreased in males [42]. Although diabetes is often accompanied by hyperlipidaemia [2], TG is associated with a decreased risk of diabetes when increased genetic susceptibility is considered [6]. As a potential predictive marker of insulin resistance (IR), the TG/HDL-C ratio is related to diabetes mellitus [5]. However, a previous study reported that no differences in TG/HDL-C were found between obese normal glucose-tolerant individuals and patients with type 2 diabetes [43]. Interestingly, the combination of genetic variations and diabetes mellitus was demonstrated to be a predictor of TG and TG/HDL-C only in the female subjects (Table 6). Thus, the gender-dependent associations with the combination of genetic variations found in the present study potentially provide an additional explanation for the inconsistent changes in TG and other indicators related to diabetes mellitus and provide new ideas to target blood lipids clinically.

### Strengths and limitations

The current study is the first to assess the correlations between the *ACE I/D* variant and the *AGTR1* rs5182 polymorphism both independently and synergistically with diabetes mellitus in a Chinese elderly population. A potential limitation of the current study was that serum ACE levels were not detected. However, the combination of different genetic variations was taken into consideration. Moreover, analysis of the younger Chinese population with diabetes is highly recommended in future studies because of the elevated prevalence of diabetes in elderly subjects [44]. Additionally, given the diverse effects of different antidiabetic and lipid-lowering drugs on metabolism [45], the identification of the associations among specific medications, diabetes and genetic backgrounds in China will be valuable.

## Conclusions

The findings of the present study suggest potential interactions among gender, *ACE I/D*, *AGTR1* rs5182 and diabetes mellitus in terms of lipid and lipid ratios, especially in terms of TG levels and the TG/HDL-C ratio. This information provides possible explanations for the contradictory associations between diabetes mellitus and lipid metabolism. Furthermore, in elderly Chinese females, TG and TG/HDL-C levels might be more susceptible to the cumulative effect of *ACE I/D* and *AGTR1* rs5182 as well as their combined effect with diabetes. Such an understanding may suggest the development of personalized treatments based on *ACE* and *AGTR1* genetic polymorphisms to lower elevated TG levels in elderly diabetic female patients that have the potential to normalize the dyslipidaemia induced by diabetes mellitus.

### Electronic supplementary material

Below is the link to the electronic supplementary material.

Supplementary Material 1

Supplementary Material 2

## Data Availability

No datasets were generated or analysed during the current study.

## Abbreviations

ACE: Angiotensin converting enzyme
AGTR1: Angiotensin type 1 receptor
ACE: Angiotensin converting enzyme gene
AGTR1: Angiotensin type 1 receptor gene
HDL-C: High-density lipoprotein cholesterol
LDL-C: Low-density lipoprotein cholesterol
ApoA: Apolipoprotein A
ApoB: Apolipoprotein B
TC: Total cholesterol
TG: Triglycerides
RAS: Renin-Angiotensin System
SDs: Standard deviations
RAR: Retinoic acid receptor
RXR: Retinoid X receptor
PPAR: Peroxisome proliferator-activated receptor
CRBP1: Cellular retinol-binding protein 1
