# Supplementary material for: Associations of ACE I/D and AGTR1 rs5182 polymorphisms with diabetes and their effects on lipids in an elderly Chinese population
Source: Lipids Health Dis. 2024 Jul 30;23:231. doi: 10.1186/s12944-024-02222-w (PMC11290002; doi:10.1186/s12944-024-02222-w)
Supplement: Supplementary file 2 — Supplementary Material 2 [file 12944_2024_2222_MOESM2_ESM.pdf]

SPRINGER NATURE
Author Services

Editing Certificate

This document certifies that the manuscript

Associations of ACE I/D and AGTR1 rs5182 polymorphisms with diabetes and their effects on lipids in Chinese elderly population

prepared by the authors

Jun Yi Liu, Yan Zhi Yi, Qi Wei Guo, Ke Xin Jia, Xue Cheng Li, Jia Jing Cai, Yi Lin Shen, Guo Ming Su, Xu Chen, Xing Yu Zhang, Ding Zhi Fang, Hao Hong*, and Jia Lin*

was edited for proper English language, grammar, punctuation, spelling, and overall style by one or more of the highly qualified native English speaking editors at SNAS.

This certificate was issued on **July 17, 2024** and may be verified on the SNAS website using the verification code **C6F8-C415-7083-4681-E7FD**.

Neither the research content nor the authors' intentions were altered in any way during the editing process. Documents receiving this certification should be English-ready for publication; however, the author has the ability to accept or reject our suggestions and changes. To verify the final SNAS edited version, please visit our verification page at secure.authorservices.springernature.com/certificate/verify.
If you have any questions or concerns about this edited document, please contact SNAS at support@as.springernature.com.

SNAS provides a range of editing, translation, and manuscript services for researchers and publishers around the world.
For more information about our company, services, and partner discounts, please visit authorservices.springernature.com.
